# Triclinic form of 1,2,4,5-tetra­cyclo­hexyl­benzene

**DOI:** 10.1107/S1600536807067578

**Published:** 2008-01-04

**Authors:** Joel T. Mague, Lisa Linhardt, Iliana Medina, Daniel J. Sattler, Mark J. Fink

**Affiliations:** a Department of Chemistry, Tulane University, New Orleans, LA 70118, USA

## Abstract

The mol­ecule of the title compound, C_30_H_46_, has a crystallographically imposed inversion center and the cyclo­hexyl groups are oriented with their methine H atoms pointing towards one another (H⋯H = 2.04 Å).

## Related literature

For related structures, see: Mague *et al.* (2008*a* ▶,*b* ▶).
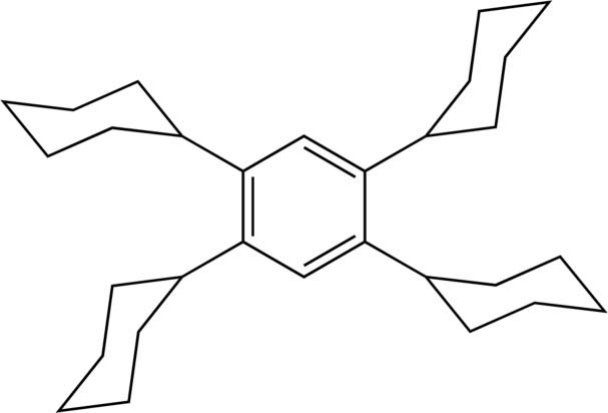


## Experimental

### 

#### Crystal data

C_30_H_46_
*M* *_r_* = 406.67
Triclinic, 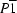

*a* = 6.014 (1) Å
*b* = 10.001 (1) Å
*c* = 10.513 (2) Å
α = 91.164 (2)°
β = 94.815 (2)°
γ = 106.336 (3)°
*V* = 604.01 (16) Å^3^
*Z* = 1
Mo *K*α radiation
μ = 0.06 mm^−1^
*T* = 100 (2) K
0.20 × 0.13 × 0.10 mm

#### Data collection

Bruker SMART APEX CCD area-detector diffractometer
Absorption correction: multi-scan (*SADABS*; Sheldrick, 2002 ▶) *T*_min_ = 0.940, *T*_max_ = 0.993
4730 measured reflections
2363 independent reflections
1862 reflections with *I* > 2σ(*I*)
*R*_int_ = 0.023

#### Refinement

*R*[*F* ^2^ > 2σ(*F* ^2^)] = 0.057
*wR*(*F* ^2^) = 0.147
*S* = 1.03
2363 reflections
136 parameters
H-atom parameters constrained
Δρ_max_ = 0.44 e Å^−3^
Δρ_min_ = −0.26 e Å^−3^

### 

Data collection: *SMART* (Bruker, 2000 ▶); cell refinement: *SAINT-Plus* (Bruker, 2004 ▶); data reduction: *SAINT-Plus* program(s) used to solve structure: *SHELXS97* (Sheldrick, 1997 ▶); program(s) used to refine structure: *SHELXL97* (Sheldrick, 1997 ▶); molecular graphics: *SHELXTL* (Bruker, 2000 ▶); software used to prepare material for publication: *SHELXTL*.

## Supplementary Material

Crystal structure: contains datablocks I, global. DOI: 10.1107/S1600536807067578/gk2128sup1.cif

Structure factors: contains datablocks I. DOI: 10.1107/S1600536807067578/gk2128Isup2.hkl

Additional supplementary materials:  crystallographic information; 3D view; checkCIF report

## Figures and Tables

**Fig. 1.**
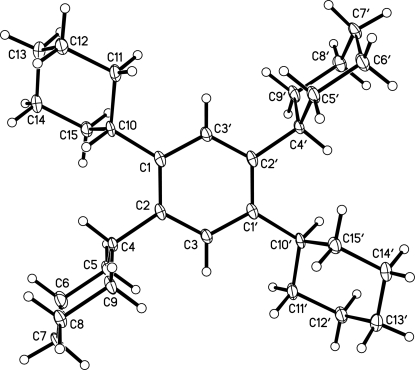
Molecular structure of the title compound. Displacement ellipsoids are drawn at the 50% probability level and H-atoms are represented by spheres of arbitrary radius. Primed atoms are related to unprimed atoms by the symmetry operation -x, 1 - y, -z.

## supplementary crystallographic information

### Comment

Crystallization of 1,2,4,5-tetracylohexylbenzene (C_30_H_46_) from hot methylcyclohexane forms colorless needle-shaped crystals together with a smaller quantity having a distinctly different block-shaped morphology. Many of the needles appear twinned but a fragment cut from a larger needle proved to be single and to be a triclinic modification. The molecule has crystallographically imposed centrosymmetry with the cyclohexyl rings adopting the chair conformation and oriented with their methine hydrogen atoms pointed towards one another (H4···H10 distance 2.04 Å) as is the case in the monoclinic modification (Mague *et al.*, 2008*a*). Again, there are very few close contacts between the *ortho*-disposed cyclohexyl rings, the shortest being H4···H15*b* (2.30 Å). Additional short contacts are H3···H9b (2.28 Å) and H3···H11*a*' (2.14 Å). The plane defined by the atoms C5, C6, C8, C9 ("seat" of the chair) is inclined to the plane of the aromatic ring by 87.3 (2)° while that for the other cyclohexyl ring (C11, C12, C14, C15) is inclined at an angle of only 49.4 (2)°, a much greater disparity in orientation than observed in the monoclinic modification. Another difference is the absence of any significant C—H···π interactions in the triclinic form.

### Experimental

The title compound was prepared by the literature method (Mague *et al.*, 2008*a*)

### Refinement

H-atoms were placed in calculated positions (C–H = 0.95 - 0.98 Å) and refined as riding on their carriers with isotropic displacement parameters 1.2 - 1.5 times those of the attached carbon atoms.

### Crystal data

**Table:**

C_30_H_46_	*Z* = 1
*M**_r_* = 406.67	*F*_000_ = 226
Triclinic, *P*1	*D*_x_ = 1.118 Mg m^−^^3^
Hall symbol: -P 1	Mo *K*α radiation λ = 0.71073 Å
*a* = 6.014 (1) Å	Cell parameters from 2522 reflections
*b* = 10.001 (1) Å	θ = 2.8–28.3º
*c* = 10.513 (2) Å	µ = 0.06 mm^−^^1^
α = 91.164 (2)º	*T* = 100 (2) K
β = 94.815 (2)º	Plate, colorless
γ = 106.336 (3)º	0.20 × 0.13 × 0.10 mm
*V* = 604.01 (16) Å^3^	

### Data collection

**Table:**

Bruker SMART APEX CCD area-detector diffractometer	2363 independent reflections
Radiation source: fine-focus sealed tube	1862 reflections with *I* > 2σ(*I*)
Monochromator: graphite	*R*_int_ = 0.023
Detector resolution: 0 pixels mm^-1^	θ_max_ = 26.0º
*T* = 100(2) K	θ_min_ = 2.0º
ω scans	*h* = −7→7
Absorption correction: multi-scan(SADABS; Sheldrick, 2002)	*k* = −12→12
*T*_min_ = 0.940, *T*_max_ = 0.993	*l* = −12→12
4730 measured reflections	

### Refinement

**Table:**

Refinement on *F*^2^	Secondary atom site location: difference Fourier map
Least-squares matrix: full	Hydrogen site location: inferred from neighbouring sites
*R*[*F*^2^ > 2σ(*F*^2^)] = 0.057	H-atom parameters constrained
*wR*(*F*^2^) = 0.147	*w* = 1/[σ^2^(*F*_o_^2^) + (0.0804*P*)^2^ + 0.1811*P*] where *P* = (*F*_o_^2^ + 2*F*_c_^2^)/3
*S* = 1.03	(Δ/σ)_max_ < 0.001
2363 reflections	Δρ_max_ = 0.44 e Å^−^^3^
136 parameters	Δρ_min_ = −0.25 e Å^−^^3^
Primary atom site location: structure-invariant direct methods	Extinction correction: none

### Special details

**Table:**

Experimental. The diffraction data were collected in three sets of 606 frames (ω scans, 0.3°/scan) at φ settings of 0, 120 and 240°.
Geometry. All e.s.d.'s (except the e.s.d. in the dihedral angle between two l.s. planes) are estimated using the full covariance matrix. The cell e.s.d.'s are taken into account individually in the estimation of e.s.d.'s in distances, angles and torsion angles; correlations between e.s.d.'s in cell parameters are only used when they are defined by crystal symmetry. An approximate (isotropic) treatment of cell e.s.d.'s is used for estimating e.s.d.'s involving l.s. planes.
Refinement. Refinement of *F*^2^ against ALL reflections. The weighted *R*-factor *wR* and goodness of fit *S* are based on *F*^2^, conventional *R*-factors *R* are based on *F*, with *F* set to zero for negative *F*^2^. The threshold expression of *F*^2^ > σ(*F*^2^) is used only for calculating *R*-factors(gt) *etc*. and is not relevant to the choice of reflections for refinement. *R*-factors based on *F*^2^ are statistically about twice as large as those based on *F*, and *R*- factors based on ALL data will be even larger. H-atoms were placed in calculated positions (C—H = 0.95 - 0.98 Å) and included as riding contributions with isotropic displacement parameters 1.2 - 1.5 times those of the attached carbon atoms.

### Fractional atomic coordinates and isotropic or equivalent isotropic displacement parameters (Å^2^)

**Table:**

	*x*	*y*	*z*	*U*_iso_*/*U*_eq_	
C1	0.1622 (3)	0.08720 (17)	0.92456 (15)	0.0140 (4)	
C2	0.0316 (3)	−0.04621 (17)	0.87681 (15)	0.0141 (4)	
C3	−0.1264 (3)	−0.12910 (17)	0.95305 (15)	0.0147 (4)	
H3	−0.2148	−0.2192	0.9199	0.018*	
C4	0.0526 (3)	−0.10585 (17)	0.74528 (15)	0.0147 (4)	
H4	0.1660	−0.0318	0.7022	0.018*	
C5	0.1482 (3)	−0.23296 (18)	0.75408 (16)	0.0179 (4)	
H5A	0.3031	−0.2056	0.8033	0.021*	
H5B	0.0431	−0.3065	0.8002	0.021*	
C6	0.1695 (3)	−0.29087 (19)	0.62131 (16)	0.0207 (4)	
H6A	0.2875	−0.2207	0.5789	0.025*	
H6B	0.2235	−0.3754	0.6301	0.025*	
C7	−0.0612 (3)	−0.32752 (18)	0.53844 (16)	0.0211 (4)	
H7A	−0.1747	−0.4051	0.5756	0.025*	
H7B	−0.0386	−0.3589	0.4518	0.025*	
C8	−0.1570 (3)	−0.20179 (19)	0.52917 (16)	0.0201 (4)	
H8A	−0.3118	−0.2296	0.4798	0.024*	
H8B	−0.0522	−0.1283	0.4829	0.024*	
C9	−0.1790 (3)	−0.14328 (18)	0.66175 (15)	0.0178 (4)	
H9A	−0.2333	−0.0590	0.6525	0.021*	
H9B	−0.2969	−0.2133	0.7043	0.021*	
C10	0.3406 (3)	0.18370 (17)	0.84830 (15)	0.0146 (4)	
H10	0.2704	0.1748	0.7578	0.018*	
C11	0.4030 (3)	0.33832 (18)	0.88976 (16)	0.0178 (4)	
H11A	0.4788	0.3524	0.9782	0.021*	
H11B	0.2591	0.3680	0.8887	0.021*	
C12	0.5673 (3)	0.42760 (18)	0.80049 (16)	0.0190 (4)	
H12A	0.4885	0.4178	0.7128	0.023*	
H12B	0.6075	0.5270	0.8296	0.023*	
C13	0.7888 (3)	0.38188 (18)	0.79954 (17)	0.0197 (4)	
H13A	0.8902	0.4373	0.7386	0.024*	
H13B	0.8746	0.4000	0.8856	0.024*	
C14	0.7330 (3)	0.22785 (18)	0.76158 (16)	0.0184 (4)	
H14A	0.8788	0.1998	0.7672	0.022*	
H14B	0.6637	0.2117	0.6718	0.022*	
C15	0.5639 (3)	0.13813 (18)	0.84782 (16)	0.0166 (4)	
H15A	0.6398	0.1461	0.9360	0.020*	
H15B	0.5238	0.0392	0.8174	0.020*	

### Atomic displacement parameters (Å^2^)

**Table:**

	*U*^11^	*U*^22^	*U*^33^	*U*^12^	*U*^13^	*U*^23^
C1	0.0186 (9)	0.0177 (9)	0.0079 (8)	0.0082 (7)	0.0026 (6)	0.0007 (6)
C2	0.0180 (9)	0.0197 (9)	0.0065 (8)	0.0083 (7)	0.0020 (6)	0.0006 (6)
C3	0.0197 (9)	0.0159 (9)	0.0085 (8)	0.0046 (7)	0.0031 (6)	−0.0002 (6)
C4	0.0219 (9)	0.0135 (8)	0.0084 (8)	0.0031 (7)	0.0066 (7)	−0.0004 (6)
C5	0.0230 (9)	0.0215 (9)	0.0100 (8)	0.0071 (7)	0.0044 (7)	−0.0016 (7)
C6	0.0276 (10)	0.0217 (10)	0.0151 (9)	0.0092 (8)	0.0076 (7)	−0.0024 (7)
C7	0.0317 (11)	0.0208 (9)	0.0101 (8)	0.0051 (8)	0.0067 (7)	−0.0047 (7)
C8	0.0274 (10)	0.0239 (10)	0.0077 (8)	0.0047 (8)	0.0023 (7)	−0.0005 (7)
C9	0.0254 (10)	0.0220 (9)	0.0078 (8)	0.0091 (7)	0.0043 (7)	−0.0002 (7)
C10	0.0194 (9)	0.0187 (9)	0.0061 (8)	0.0051 (7)	0.0046 (6)	0.0002 (6)
C11	0.0220 (9)	0.0197 (9)	0.0138 (8)	0.0071 (7)	0.0093 (7)	0.0008 (7)
C12	0.0264 (10)	0.0176 (9)	0.0143 (9)	0.0066 (7)	0.0083 (7)	0.0008 (7)
C13	0.0215 (9)	0.0221 (10)	0.0155 (9)	0.0045 (7)	0.0081 (7)	0.0027 (7)
C14	0.0205 (9)	0.0250 (10)	0.0122 (8)	0.0087 (7)	0.0070 (7)	0.0025 (7)
C15	0.0215 (9)	0.0179 (9)	0.0112 (8)	0.0059 (7)	0.0045 (7)	0.0017 (7)

### Geometric parameters (Å, °)

**Table:**

C1—C3^i^	1.397 (2)	C8—H8B	0.9900
C1—C2	1.402 (2)	C9—H9A	0.9900
C1—C10	1.523 (2)	C9—H9B	0.9900
C2—C3	1.395 (2)	C10—C11	1.530 (2)
C2—C4	1.524 (2)	C10—C15	1.535 (2)
C3—C1^i^	1.397 (2)	C10—H10	1.0000
C3—H3	0.9500	C11—C12	1.532 (2)
C4—C9	1.529 (2)	C11—H11A	0.9900
C4—C5	1.537 (2)	C11—H11B	0.9900
C4—H4	1.0000	C12—C13	1.526 (2)
C5—C6	1.529 (2)	C12—H12A	0.9900
C5—H5A	0.9900	C12—H12B	0.9900
C5—H5B	0.9900	C13—C14	1.519 (2)
C6—C7	1.522 (3)	C13—H13A	0.9900
C6—H6A	0.9900	C13—H13B	0.9900
C6—H6B	0.9900	C14—C15	1.529 (2)
C7—C8	1.525 (2)	C14—H14A	0.9900
C7—H7A	0.9900	C14—H14B	0.9900
C7—H7B	0.9900	C15—H15A	0.9900
C8—C9	1.531 (2)	C15—H15B	0.9900
C8—H8A	0.9900		
			
C3^i^—C1—C2	117.64 (15)	C8—C9—H9A	109.3
C3^i^—C1—C10	120.34 (15)	C4—C9—H9B	109.3
C2—C1—C10	122.00 (14)	C8—C9—H9B	109.3
C3—C2—C1	118.62 (14)	H9A—C9—H9B	107.9
C3—C2—C4	117.96 (14)	C1—C10—C11	114.86 (13)
C1—C2—C4	123.43 (14)	C1—C10—C15	111.62 (13)
C2—C3—C1^i^	123.74 (16)	C11—C10—C15	109.51 (14)
C2—C3—H3	118.1	C1—C10—H10	106.8
C1^i^—C3—H3	118.1	C11—C10—H10	106.8
C2—C4—C9	111.87 (13)	C15—C10—H10	106.8
C2—C4—C5	111.95 (13)	C10—C11—C12	110.82 (13)
C9—C4—C5	110.22 (14)	C10—C11—H11A	109.5
C2—C4—H4	107.5	C12—C11—H11A	109.5
C9—C4—H4	107.5	C10—C11—H11B	109.5
C5—C4—H4	107.5	C12—C11—H11B	109.5
C6—C5—C4	111.24 (14)	H11A—C11—H11B	108.1
C6—C5—H5A	109.4	C13—C12—C11	110.19 (14)
C4—C5—H5A	109.4	C13—C12—H12A	109.6
C6—C5—H5B	109.4	C11—C12—H12A	109.6
C4—C5—H5B	109.4	C13—C12—H12B	109.6
H5A—C5—H5B	108.0	C11—C12—H12B	109.6
C7—C6—C5	111.78 (14)	H12A—C12—H12B	108.1
C7—C6—H6A	109.3	C14—C13—C12	111.15 (15)
C5—C6—H6A	109.3	C14—C13—H13A	109.4
C7—C6—H6B	109.3	C12—C13—H13A	109.4
C5—C6—H6B	109.3	C14—C13—H13B	109.4
H6A—C6—H6B	107.9	C12—C13—H13B	109.4
C6—C7—C8	110.76 (14)	H13A—C13—H13B	108.0
C6—C7—H7A	109.5	C13—C14—C15	111.44 (14)
C8—C7—H7A	109.5	C13—C14—H14A	109.3
C6—C7—H7B	109.5	C15—C14—H14A	109.3
C8—C7—H7B	109.5	C13—C14—H14B	109.3
H7A—C7—H7B	108.1	C15—C14—H14B	109.3
C7—C8—C9	111.34 (14)	H14A—C14—H14B	108.0
C7—C8—H8A	109.4	C14—C15—C10	111.17 (14)
C9—C8—H8A	109.4	C14—C15—H15A	109.4
C7—C8—H8B	109.4	C10—C15—H15A	109.4
C9—C8—H8B	109.4	C14—C15—H15B	109.4
H8A—C8—H8B	108.0	C10—C15—H15B	109.4
C4—C9—C8	111.79 (14)	H15A—C15—H15B	108.0
C4—C9—H9A	109.3		
			
C3^i^—C1—C2—C3	0.3 (3)	C2—C4—C9—C8	179.42 (14)
C10—C1—C2—C3	179.24 (15)	C5—C4—C9—C8	−55.33 (18)
C3^i^—C1—C2—C4	−179.88 (15)	C7—C8—C9—C4	55.87 (19)
C10—C1—C2—C4	−0.9 (2)	C3^i^—C1—C10—C11	−21.8 (2)
C1—C2—C3—C1^i^	−0.3 (3)	C2—C1—C10—C11	159.29 (15)
C4—C2—C3—C1^i^	179.86 (15)	C3^i^—C1—C10—C15	103.66 (18)
C3—C2—C4—C9	59.29 (19)	C2—C1—C10—C15	−75.29 (19)
C1—C2—C4—C9	−120.57 (17)	C1—C10—C11—C12	−175.25 (14)
C3—C2—C4—C5	−65.00 (19)	C15—C10—C11—C12	58.25 (18)
C1—C2—C4—C5	115.15 (17)	C10—C11—C12—C13	−58.39 (19)
C2—C4—C5—C6	−179.65 (14)	C11—C12—C13—C14	56.47 (18)
C9—C4—C5—C6	55.15 (18)	C12—C13—C14—C15	−55.38 (19)
C4—C5—C6—C7	−55.97 (19)	C13—C14—C15—C10	55.66 (19)
C5—C6—C7—C8	55.61 (19)	C1—C10—C15—C14	175.04 (13)
C6—C7—C8—C9	−55.23 (19)	C11—C10—C15—C14	−56.63 (17)

Symmetry codes: (i) −*x*, −*y*, −*z*+2.
